# Oviduct and endometrial epithelium improve *in vitro* produced bovine embryo developmental kinetics

**DOI:** 10.1530/REP-24-0008

**Published:** 2024-04-17

**Authors:** L Kirsten Senn, Katheryn D Peterson, J Lannett Edwards, Rebecca R Payton, Daniel J Mathew

**Affiliations:** 1 Department of Animal Science, University of Tennessee Institute of Agriculture, University of Tennessee, Knoxville, Tennessee, USA

## Abstract

**In brief:**

Standard *in vitro* produced (IVP) bovine embryo culture media limit embryonic development. Culturing IVP bovine embryos in standard IVP bovine embryo culture media conditioned with oviduct and/or endometrial cells improves blastocyst formation and reduces the time to formation.

**Abstract:**

*In vitro* embryo production in cattle greatly impacts blastomere biochemistry, embryo rate of development and pre- and post-transfer survival. *In vivo*, the bovine embryo migrates through the oviduct isthmus before entering the uterus on approximately day 4 of development where it remains unattached within the uterine lumen until day 20 of gestation. During this time, the embryo is sequentially exposed to oviduct followed by endometrial secretions that support embryonic development. Considering this, we tested the effect of culturing *in vitro* produced (IVP) bovine embryos sequentially in oviduct epithelial- (OEp; days 1–3) followed by endometrial epithelial- (EEp) or EEp and fibroblast cell (EEp/F; days 4–8)-conditioned media on embryonic development using a time-lapse monitoring system. Compared to control, culturing IVP embryos in EEp- or EEp/F-conditioned media without prior culture in OEp-conditioned media increased blastocyst formation (*P* < 0.05) and reduced the time to blastocyst formation (*P* < 0.05). Culturing IVP bovine embryos in OEp-conditioned media followed by EEp- or EEp/F-conditioned media, however, had the greatest impact on embryo developmental kinetics and increased morula and blastocyst formation (*P* < 0.05) and reduced time to formation (*P* < 0.05). Day 8 blastocyst cell numbers, diameter and quality were not significantly different, although, blastocyst quality scores were less (indicative of better quality) for all cell-conditioned media compared to control. In conclusion, IVP bovine embryo development may be improved using a sequential embryo culture system involving bovine oviduct followed by endometrial cell-conditioned media.

## Introduction

*In vitro* produced (IVP) cattle embryos are a valuable resource to study early embryonic development in mammals and improve beef and dairy farm herd genetics for various food production traits. For these reasons and others, IVP cattle embryos have become more popular and between the years 2020 and 2021, production and transfer of IVP cattle embryos increased by over 30% and accounted for over 70% of all transferrable cattle embryos worldwide (Viana 2022). A considerable limitation associated with the technology, however, is the rate of embryonic development and survival post-transfer associated with IVP cattle embryos. Even in the best laboratories, IVP bovine embryo blastocyst formation rates rarely exceed 40% and pregnancy rates associated with IVP cattle embryos are approximately 50% lower than females that undergo artificial insemination (Lonergan & Fair 2008, Ealy *et al.* 2019). Alarmingly, based on a collection of studies, pregnancies associated with IVP cattle embryos failed to reach term in 59% to 85% of recipients (Ealy *et al.* 2019).

Compared to *in vivo* derived embryos, studies suggest that IVP cattle embryos have an altered biochemistry including altered lipid content, epigenome, and transcriptome as well as an abnormal inner cell mass (ICM)-to-trophectoderm (TE) ratio (Iwasaki *et al.* 1990, Rizos *et al.* 2002, 2008, Driver *et al.* 2012). If the IVP embryo can overcome this, these alterations likely manifest into abnormal juxtracrine and paracrine communication with the dam as endometrium cultured with IVP cattle embryos has an altered transcriptome (Mansouri-Attia *et al.* 2009, Biase *et al.* 2016, Mathew *et al.* 2019). IVP embryo biochemical and signaling alterations may be the result of two major limitations related to *in vitro* embryo production: 1) developmental stage or quality of the oocyte used to generate IVP cattle embryos and 2) the *in vitro* culture conditions including suboptimal embryo culture media (Rizos *et al.* 2002, Biase *et al.* 2016).

During *in vivo* development in cattle*,* oocyte fertilization occurs in the ampulla–isthmus junction of the oviduct followed by initial embryonic development within the isthmus. There, oviduct epithelial cell (OEp) secretions support sperm–egg binding, embryo first cleavage and embryonic genome activation (Tesarík *et al.* 1988, Suarez 2007, Avilés *et al.* 2010, Gad *et al.* 2012). On approximately day 4 of development, the oviduct ciliated epithelial cells and oviduct smooth muscle assist embryo migration into the uterine lumen where it is supported by progesterone (P4) driven secretions from the endometrial mucosa collectively referred to as histotroph (Mathew *et al.* 2022). These secretions are largely from the endometrial epithelial cells (EEp), particularly, glandular epithelium (GE) within the intercaruncular regions. However, the underlying endometrial stromal fibroblast cells (EF) likely contribute to histotroph via molecule transport through the simple epithelial barrier and paracrine communication with EEp and GE that support optimal epithelial secretions (Chaney *et al.* 2021, Mathew *et al.* 2022).

The EEp secretions support early embryonic development and are essential for conceptus elongation in cattle. The bovine conceptus maintains a protracted period of development and will remain unattached from the endometrium until approximately day 20 of gestation (Mathew *et al.* 2022). Critical developmental processes in ungulates such as conceptus elongation do not occur *in vitro.* Further, endometrium lacking uterine glands does not support pregnancy (Gray *et al.* 2001). During a recent study, we evaluated the effects of using bovine EEp and EF to condition a standard cell culture medium to support IVP bovine embryonic development (Oliver *et al.* 2023). When compared to non-cell-conditioned medium, culturing embryos in the EEp- or EF-conditioned media from days 4 to 8 of development resulted in a greater number of blastocysts and affected blastocyst gene expression (Oliver *et al.* 2023). Based on this information, we designed a follow-up study to investigate the impact of culturing IVP bovine embryos sequentially in a standard embryo culture medium conditioned by OEp followed by EF and or EEp using the MIRI TL6, an advanced embryo monitoring system. We hypothesize that bovine oviduct and endometrial cell secretions, when available in sequence during embryonic development, could be used to improve IVP bovine embryo survival and developmental kinetics.

## Materials and methods

### Tissue collection and oviduct cell isolation

**Figure 1:**
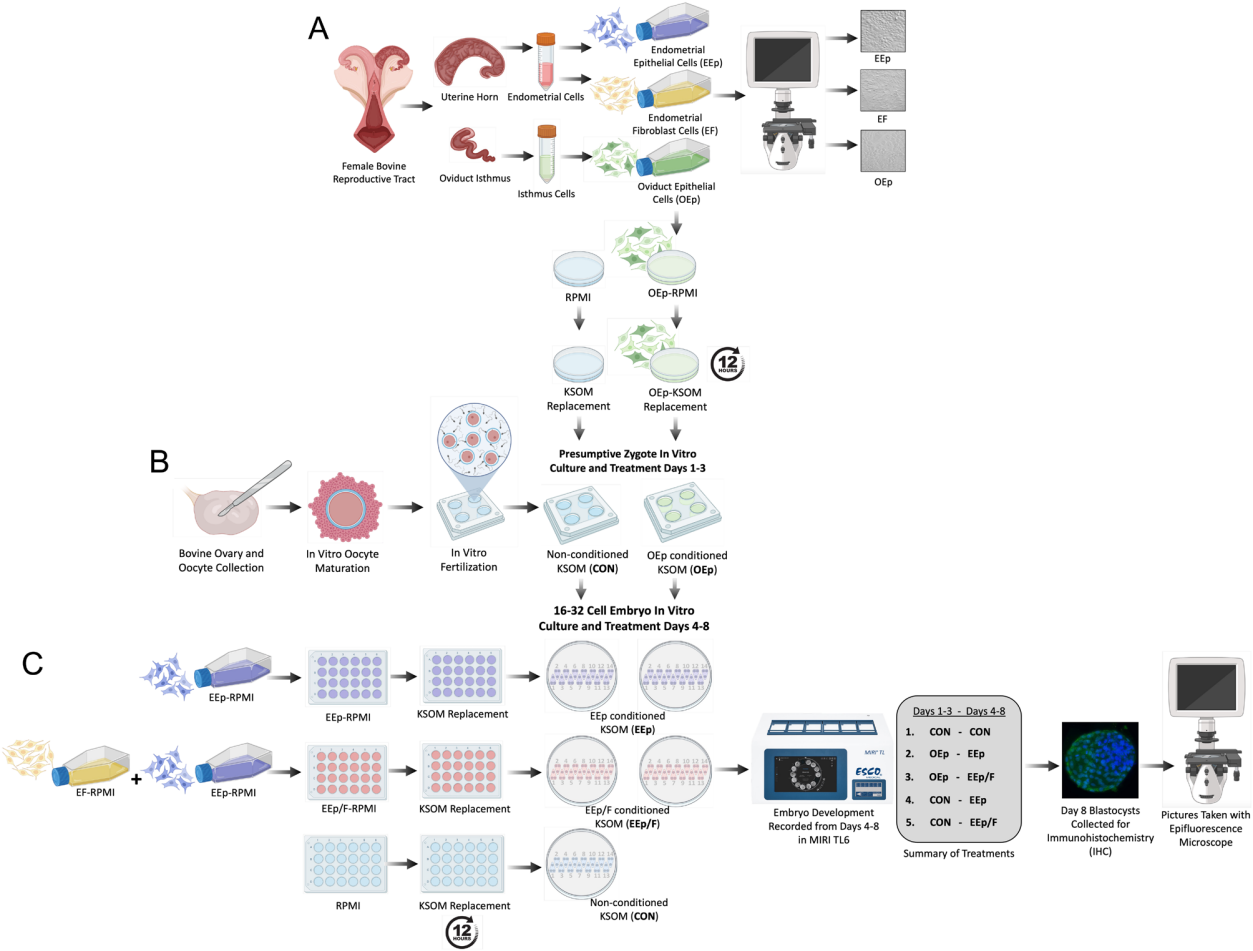
Graphic illustrating the study experimental design. (A) For each female bovine reproductive tract (*n* = 6), the uterine horn and oviduct isthmus ipsilateral to the corpus luteum were dissected and used to isolate endometrial epithelial (EEp) and fibroblast cells (EF) and oviduct epithelial cells (OEp), respectively. (B) Approximately 2 days before bovine presumptive zygotes (PZ) were to be cultured in conditioned medium, OEp were plated into a petri dish. A second petri dish received culture medium (Roswell Park Memorial Institute; RPMI) without cells. Once OEp reached confluency, the culture medium was removed from both dishes and replaced with fresh potassium simplex optimized medium (KSOM). The KSOM was allowed to incubate in both dishes for 12 h, producing OEp-conditioned medium and non-cell-conditioned (CON) medium. Bovine PZ, produced using standard *in vitro* embryo production methods, were then cultured (group culture; 20–35) with the CON (*n* = 807) or OEp (*n* = 549) medium in four-well plates from days 1 to 3 of development. On day 3, PZ rate of cleavage and formation of 8–16 cells were measured manually. (C) Approximately 2 days before 16–32-cell embryos were to be transferred into a second conditioned medium, EEp or EEp and EF were plated into 24-well plates, the latter of which resulted in EEp/F cocultures. A third 24-well plate received RPMI without cells. Once the cells reached confluency, medium was removed from all dishes and replaced with fresh KSOM. The KSOM was allowed to incubate in the 24-well plates for 12 h producing CON, EEp, and EEp/F-conditioned media. On day 4 of development, 16–32-cell embryos from the previous CON medium were equally transferred into the new CON, EEp, EEp/F medium in MIRI culture coins (7–14/coin; *n* = 77). Similarly, 16–32-cell embryos from the previous OEp medium were equally transferred into the EEp or EEp/F medium in MIRI culture coins (7–14/coin; *n* = 77). The embryos were then monitored continuously from days 4 to 8 of development in the MIRI time lapse 6 (TL6) embryo incubator. On day 8, blastocysts were removed from the MIRI and stained for trophoblast marker caudal-type homeobox 2, and cell nuclei with 4′,6-diamidino-2-phenylindole in preparation of TE and ICM cell counts. MIRI time lapse videos of developing embryos were used to measure embryo structure formation (compact morula and early, normal, and expanded blastocyst) and time to structure formation as well as day 8 expanded blastocyst diameter and quality. Graphic was created using illustrations from BioRender.com.

**Figure 2:**
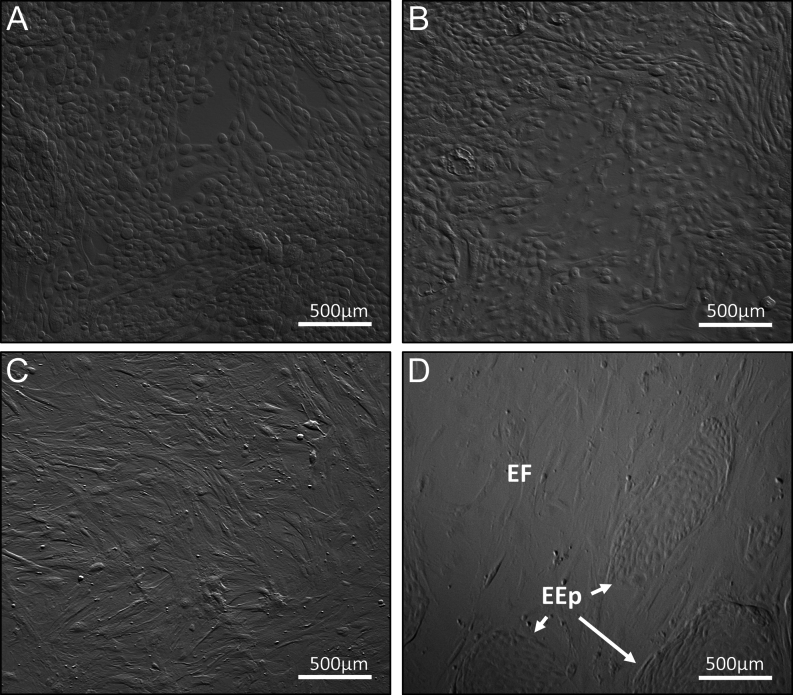
Images of confluent female reproductive tract cells used to condition potassium simplex optimized medium (KSOM). D, Image of cocultured endometrial fibroblast (EF) and epithelial cells (EEp) used to prepare the EEp/F-conditioned KSOM. A, oviduct epithelial cells (OEp); B, endometrial epithelial cells (EEp), C, endometrial fibroblast cells (EF). Bar = 500 µm.

This study did not involve live animals. Rather, all animal derived tissues were opportunistically collected from a USDA-inspected abattoir. Female bovine reproductive tracts (*n* = 6), with no noticeable infection or pregnancy, were collected during the early to mid-luteal phase of the estrous cycle (corpus luteum (CL) stage II or III) as described by Ireland *et al.* (1980). The tracts were transported to laboratory (45 min), rinsed with tap water, and sprayed with 70% ethyl alcohol. The oviduct was then dissected from the uterine horn ipsilateral to the CL, placed on ice and OEp isolated from the isthmus region of the oviduct as previously described by García *et al.* (2017) but with slight modifications. Briefly, the isthmus was dissected away from the reproductive tract, cut into 1 in sections in a petri dish and OEp were expressed using forceps and a glass microscope slide. Cells aggregates were then suspended in Dulbecco’s phosphate buffered saline (DPBS, Gibco), aspirated into 15 mL conical tubes (Falcon) containing 7 mL DPBS, separated into smaller cell aggregates and single cells using a needle and syringe and centrifuged at 300 ***g*** for 10 min. After removing the supernatant, the cells were resuspended in Roswell Park Memorial Institute (RPMI, Gibco) medium containing 10% heat-inactivated fetal bovine serum (FBS; Seradigm) and 1% antibiotic antimycotic (ABAM, Gibco) (standard culture medium) and plated into T75 flasks (Greiner Bio-One). The cells were cultured at 38.5°C in 5.5% carbon dioxide (CO_2_) and atmospheric oxygen (O_2_) (standard culture conditions) for 2 weeks as previously by García *et al.* (2017) before used to condition embryo culture media (Fig. 1A, B, and 2).

### Endometrial cell isolation

Using the same reproductive tract used to isolate OEp, EEp, and EF cells were isolated from the uterine horn ipsilateral to the CL as previously described in detail by Chaney *et al.* (2021). Briefly, the uterine horn was dissected from the reproductive tract and opened longitudinally along the anti-mesometrial side. Using scissors, endometrial intercaruncular strips were dissected away from the myometrium and washed through a series of Hanks Balanced Salt Solution (HBSS; Gibco) washes including two 25 mL washes of HBSS and one 25 mL wash of HBSS containing 1% ABAM. The endometrial tissue was then enzymatically digested in HBSS solution containing collagenase type II (0.5 mg/mL; Sigma), trypsin (2.5 BAEE units/mL; Sigma), bovine serum albumin (BSA; 1 mg/mL; Sigma), and DNase 1 (0.1mg/mL; Sigma) for 1 h at 38.5°C with periodic agitation. The resulting solution was then double-filtered through a 100 µm filter over a 40 µm filter (Falcon), and the filtrate, containing mostly EF cells, was centrifuged at 700 ***g*** × 7 min. After removing the supernatant, the pellet underwent a red blood cell lysis step (addition of Milli-Q ultrapure sterile water for 30 s) before being suspended in HBSS (10% FBS) and recentrifuged at 700 ***g*** × 7 min. The final cell pellet was then suspended in 45 mL of standard culture medium and plated in three T75 flasks. To isolate and establish EEp cultures, the 40 µm filter, containing mostly endometrial epithelial tissue, was backwashed with 30 mL of standard culture media and plated into two T75 flasks. The EEp and EF cells were cultured at standard culture conditions and purified (>99%) over the course of 2 weeks by taking advantage of their differential adhesion properties and the enzymatic solution Accutase (Sigma) as previously described by Chaney *et al.* (2021). The EF were passaged approximately three times, and the EEp was passaged once during the 2-week culture period. After the 2-week period, the EEp and EF cells were used to condition the embryo culture media (Figs. 1A, B and 2).

### Preparing cell-conditioned media

Approximately, 60 h prior to embryo treatment with cell-conditioned media, OEp were seeded at a density of 0.5 ×10^6^ live cells into 35 mm petri dishes (Falcon) and EEp cells were seeded at a density of 1.0 × 10^5^ live cells into individual wells of a 24-well plate (Greiner Bio-One). A 1:1 EEp and EF combination culture (EEp/F; coculture) was also prepared by combining 0.5 × 10^5^ live EEp and 0.5 × 10^5^ live EF cells (1.0 × 10^5^ total live cells) into individual wells of a 24-well plate. Forty-eight hours later (12 h before embryo treatment), the oviduct and endometrial cells (approximately 90% confluent) were washed with 3 mL (oviduct cells) or 1 mL (endometrial cells) of DPBS (1% ABAM) followed by the addition of 3 mL (oviduct cells) or 1 mL (endometrial cells) of KSOM for 12 h (Fig. 1.1B and 1.1C) to prepare cell-conditioned media. Before being added to the cells, the KSOM was prepared fresh and supplemented with 0.5% BSA, 1% nonessential amino acids (NEAA; Sigma), and 0.05% penicillin–streptomycin (Pen-Strep; Sigma) as previously described (Biggers *et al.* 2000). The KSOM was also applied to dishes and wells without cells to create the control media (non-cell-conditioned media). These dishes and wells also received standard culture media but without cells at the time of cell plating and prior to the addition of KSOM. During KSOM conditioning (12 h), all cell dishes and plates were cultured at 38.5°C in 5.5% CO_2_ and 7% O_2_. After 12 h, the media was collected and used fresh for embryo treatments (Fig. 1A and B). Overall, the following conditioned KSOM media were created and used fresh during the study: i) non-cell-conditioned media (control; CON), ii) OEp-conditioned media, iii) EEp-conditioned media and iv) EEp/F-conditioned media. Because embryos would be cultured from day 1 to 3 with OEp-conditioned media followed by culture from day 4 to 8 with EEp or EEp/F-conditioned media (Fig. 1B and C), plating of the endometrial cells and conditioning of the media were staggered with that of the OEp for each reproductive tract/*in vitro* fertilization (IVF) repetition. Overall, a total of six female bovine reproductive tracts were used for OEp, EEp, and EF cell isolation and media conditioning. That is, one tract per oocyte/IVF repetition (six repetitions) was used during embryo treatment.

### Generating IVP bovine embryos and embryo treatments

All chemicals and reagents used for *in vitro* embryo production were purchased from MilliporeSigma and all media preparations and methods were performed as described in detail by Schrock *et al.* (2007). Briefly, ovaries collected from a local abattoir were used for oocyte collection via follicular slicing. Oocytes maintaining a compact cumulus and homogeneous ooplasm were matured for 24 h and fertilized (day 0) with frozen-thawed semen from a single bull of proven fertility following Percoll gradient centrifugation as described by Rispoli *et al.* (2011). Approximately, 16 h after fertilization (day 1), cumulus cells surrounding the presumptive zygote (PZ) were removed by vortex in HEPES-TALP containing hyaluronidase (0.3 mg/mL). The study was designed to test the effect of OEp on embryo cleavage rate by day 3 of development followed by the effect of EEp or EEp/F on embryonic structure formation between day 4 and 8 of development. Thus, to begin the experiment and for each oocyte/IVF repetition, PZ presenting even intracytoplasmic coloration were removed from the HEPES-TALP, divided, and washed twice in their respective treatments before finally added to one of the two following treatments in groups of 20–35 in Nunc (Thermofisher) four-well plates: i) 500 µL of CON or ii) 500 µL of OEp (one treatment per plate). Overall, across all repetitions, *n* = 807 (32 wells total) and *n* = 549 (22 wells total) PZ were subjected to CON and OEp treatments, respectively. The presumptive zygotes were subjected to their respective treatments at 38.5°C in 5.5% CO_2_ and 7% O_2_ (standard embryo culture conditions) from days 1 to 3 of development (72 h after fertilization).

On day 3, PZ cleavage rate was visually assessed for each well. All wells were then supplemented with 10 µL 1× basal medium eagle (BME) essential amino acids (Sigma), and embryos were placed back in the incubator to continue development until day 4. Cleavage rate (percentage) was determined by dividing the total number of cleaved embryos in each treatment well by the total number of PZs in each treatment well and multiplying by 100 (total number cleaved embryos/total number PZs × 100). Similarly, the rate of 8–16-cell embryos (percentage) was also calculated by dividing the total number of 8–16-cell embryos in each treatment well by the total number of cleaved embryos in each treatment well and multiplying by 100 (total number 8–16-cell embryos/total number embryos × 100).

On day 4, all 16-32 cell embryos, still within their treatment groups, were washed with HEPES-TALP. Embryos from the previous CON treatment were then subdivided and cultured individually from days 4–8 in one of the following three media in an embryo time lapse incubator (MIRI TL6; Esco Medical): a) 25 µL of non-cell-conditioned media (CON), b) 25 µL of EEp-conditioned media, or c) 25 µL of EEp/F-conditioned media. Each treatment corresponded to an individual MIRI culture coin with 14 wells per coin. Embryos from the previous OEp treatment were also subdivided and cultured individually in two additional culture coins containing b) 25 µL of EEp-conditioned media or c) 25 µL of EEp/F-conditioned media (Fig. 1B and C). Prior to adding the treatment media to the MIRI culture coin wells (overall, one treatment per coin, five coins total), all media (cell-conditioned and non-cell-conditioned) were supplemented with 2% BME. Before placing embryos in the MIRI incubator, embryo culture coins were overlaid with sterile mineral oil (Sigma) as suggested by the manufacturer. Depending on oocyte collection/IVF repetition, 7–14 embryos were subjected to each treatment/MIRI coin (six repetitions for a total of *n* = 77 embryos/treatment). Thus, collectively, the following embryo treatments were developed from the previous day 1–3 culture and current day 4–8 culture: i) CON-CON, ii) CON-EEp, iii) CON-EEp/F, iv) OEp-EEp, and v) OEp-EEp/F (Fig. 1C). Because of limitations related to oocyte numbers, an OEp-CON treatment was not included. From days 4 to 8 of development, the MIRI TL6 took pictures of each embryo every 5 min at seven different focal planes without disturbing the embryo culture environment (38.5°C in 5.5% CO_2_ and 7% O_2_). The pictures and videos were later used to evaluate embryo developmental kinetics (embryo structure formation rate and time to structure formation) as well as day 8 expanded blastocyst diameter and quality score (International Embryo Technology Society (IETS); quality score 1–4, excellent/good to degenerating/dead, respectively). On day 8 and after MIRI data collection, blastocysts (early, normal, and expanded) were collected and underwent immunohistochemistry for differential cell staining (ICM vs TE; Fig. 1C).

### MIRI data collection and blastocyst staining

MIRI TL6 images and time-lapse videos were used to evaluate embryo structure rate of formation (IETS stage: morula, early blastocyst, normal blastocyst, expanded blastocyst, and hatched blastocyst) as well as the time of structure formation for all structures (hours post insemination; HPI) and day 8 expanded blastocyst (grade 3) diameter at 192 HPI. Embryo stage and day 8 expanded blastocyst grade and diameter were assessed as previously described by Bó and Mapletoft (2013). After MIRI data collection, all day 8 blastocysts were stained for nuclei and the TE transcription factor caudal type homeobox 2 (CDX2) as previously described by Wooldridge *et al.* (2019) but with modifications. Briefly, blastocysts were fixed in 4% paraformaldehyde containing 0.2% polyvinylpyrrolidone for 20 min before being permeabilized with 0.25% Triton X-100 (Thermo Scientific) in DPBS. Embryos were blocked in DPBS containing 0.1% Tween 20 (PBST) and 5% BSA and then incubated overnight in 20 µL of ready-to-use mouse anti-CDX2 antibodies (BioGenex; CDX2-88). Embryos were then washed three times in wash buffer (PBST; 0.1% BSA) before being incubated in fluorescein isothiocyanate-labeled goat anti-mouse IgG Alexa Fluor 488 (Abcam) in PBST (1:500) containing 1% BSA for 1 h at RT in the dark. Embryos were then washed three times in wash buffer before being mounted on frosted glass microscope slides (Fisher) in Fluoromount-G containing 4′,6-diamidino-2-phenylindole (DAPI; ThermoScientific). An inverted microscope with epifluorescence capabilities (SMZ800 Nikon) and camera (PCO.Panda; Nikon) were used to capture fluorescent images of the stained embryos. The ImageJ computer program and cell counter plugin (NIH) were used to count the number of DAPI and CDX2 positive cells and calculate the embryo ICM to TE ratio as previously described by Xie *et al.* (2017). The number of observations for each blastocyst stage is reported in Supplementary Table 1 (see section on supplementary materials given at the end of this article).

### Statistical analysis

All data were analyzed using the statistical analysis software (SAS 9.4; SAS Institute). Normality of the data was confirmed using a Proc Univariate (Shapiro–Wilk > 0.90) and a Proc GLIMMIX was used to analyze the data. During the analysis, binomial data (MIRI -day 4–8 embryo structure formation) were indicated using the ‘dist=binomial’ statement. The average rate of PZ cleavage, embryo 8–16-cell formation, MIRI say 4–8 embryo time to structure formation, expanded blastocyst diameter and quality as well as blastocyst cell number data had a normal distribution which was indicated in Proc GLIMMIX using the ‘dist=normal’ statement (Supplementary Table 2). During the analysis, data were blocked on replicate (oocyte collection/IVF repetition) and MIRI culture coin/chamber using the ‘random’ statement.

For the MIRI day 4–8 data, three statistical analyses (SA1–3) were conducted. Statistical analysis 1 tested the hypothesis that significant differences in embryonic development between days 4 and 8 were because of culture conditions between days 1 and 3 involving OEp-conditioned media. The following three embryo culture conditions were compared: i) embryos cultured in non-cell-conditioned media from days 1 to 3 followed by non-cell-conditioned media from days 4 to 8 (CON-CON), ii) embryos cultured in OEp-conditioned media from days 1 to 3 followed by endometrial cell-conditioned media (OEp+; OEp-EEp and OEp-EEp/F data combined), and iii embryos cultured in non-cell-conditioned media followed by endometrial cell-conditioned media (CON+; CON-EEp and CON-EEp/F data combined). Statistical analysis 2 tested the hypothesis that significant differences in embryonic development existed between days 4 and 8 because of culture conditions between days 4 and 8 involving endometrial cell-conditioned media. The following three embryo culture treatments were compared: i) CON-CON, ii) embryos cultured in EEp-conditioned media from days 4 to 8 after culture in CON or OEp-conditioned media from days 1 to 3 (+EEp; CON-EEp and OEp-EEp data combined), and iii) embryos cultured in EEp/F-conditioned media from days 4 to 8 after culture in CON or OEp media (+EEp/F; CON-EEp/F and OEp-EEp/F data combined). The aim of SA3 was to determine which of the following day 1 to 8 culture conditions (sequential culture) had the greatest impact on embryonic development: i) CON-CON, ii) CON-EEp, iii) CON-EEp/F, iv) OEp-EEp, and v) OEp-EEp/F. A Tukey statement was used for comparisons during SA3. All data are presented as least squares means ± s.e. of the least squares means (LSM ± s.e.m.). Statistical significance and tendency for significance were declared at *P* ≤ 0.05 and *P* ≤ 0.10, respectively.

## Results

### Effect of OEp-conditioned medium on presumptive zygote cleavage

On day 3, compared to CON, there was no effect of OEp-conditioned KSOM on PZ cleavage (Table 1). Additionally, of the PZ that cleaved, there was no effect of OEp on the number of 8–16-cell embryos when compared to CON (Table 1).

**Table 1:** Day 3 embryo cleavage and 8–16 cell formation rate of development (percentage) after treating presumptive zygotes (PZ) from days 1 to 3 with non-conditioned (control; CON) potassium simplex optimized medium (KSOM) or oviduct epithelial cell (OEp)-conditioned KSOM. Overall number (n) of culture wells and PZ representing each treatment are also presented. Data are presented as least squares means (LSM) ± s.e. of the LSM.

	TRT		*P*
	CON	OEp	
Wells, *n*	32	22	NA
PZ, *n*	807	549	NA
Cleavage, %	64.4 ± 4.3	64.8 ± 4.4	NS
8–16 cell, %	72.6 ± 4.3	69.6 ± 4.5	NS

NA, not applicable; NS, not significant.

### Effect of cell-conditioned medium on compact morula and early blastocyst formation

**Figure 3:**
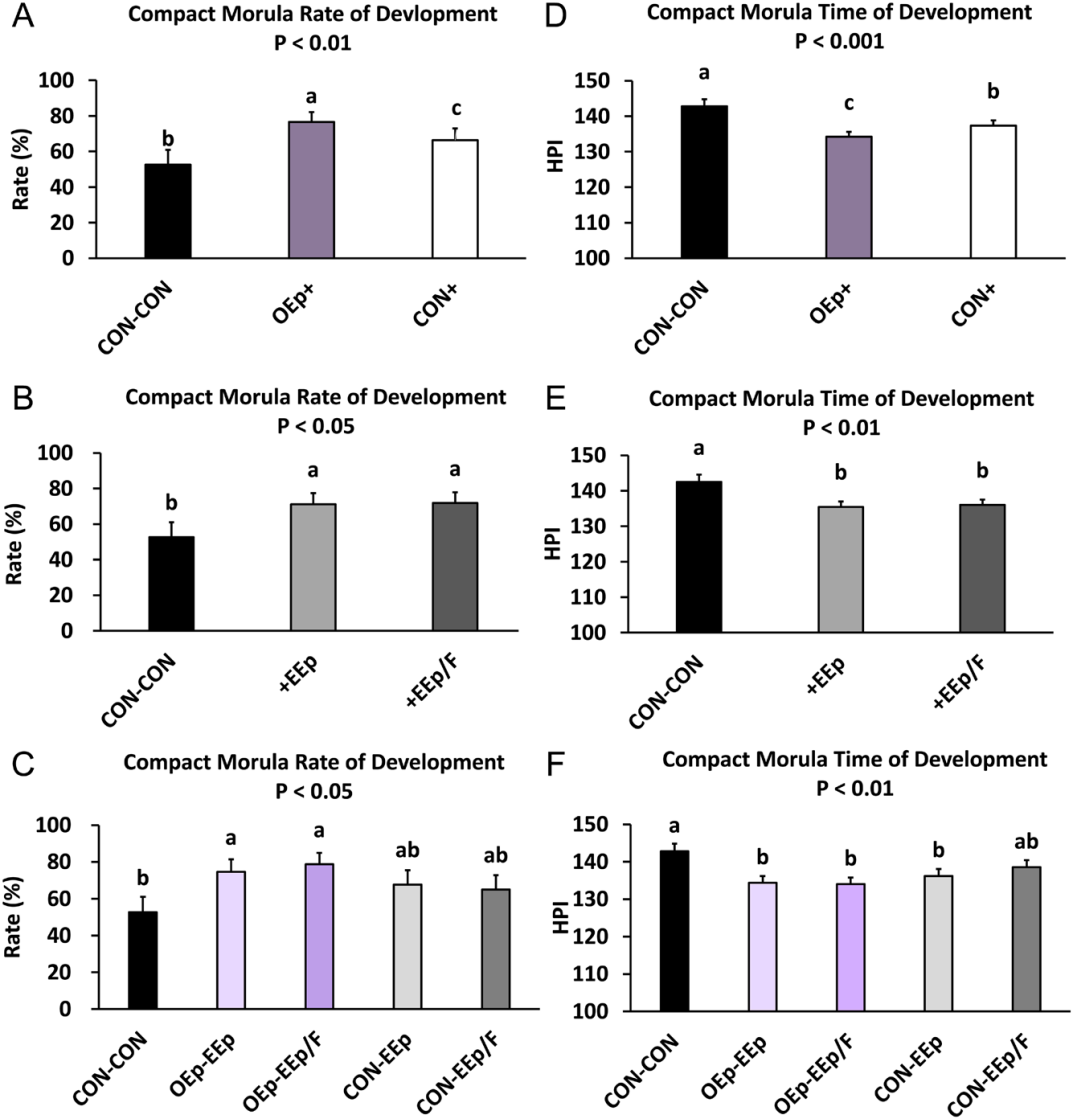
Percentage of compact morula development and time of development during sequential embryo culture in bovine reproductive cell-conditioned potassium simplex optimized medium (KSOM). (A and D; statistical analysis 1; SA1) Percentage of compact morula development and time of development (hours post-insemination; (HPI)) between days 4 and 8 for embryos sequentially cultured in non-cell-conditioned KSOM from days 1 to 3 followed by days 4 to 8 (CON-CON) and embryos cultured in oviduct epithelial cell-conditioned KSOM or CON from days 1 to 3 (OEp+ or CON+, respectively) followed by culture in endometrial epithelial (EEp) and endometrial epithelial and fibroblast cell (EEp/F; coculture) conditioned KSOM (EEp and EEp/F data combined) from days 4 to 8. (B and E; SA2) Percentage of compact morula development and time of development (HPI) between days 4 and 8 for embryos sequentially cultured in CON-CON and embryos cultured in EEp or EEp/F cell-conditioned KSOM from days 4 to 8 (+EEp or +EEp/F, respectively) after prior culture in oviduct epithelial cell-conditioned KSOM (OEp) and CON (data combined) from days 1 to 3. (C and F; SA3) Percentage of compact morula development and time of development between days 4 and 8 for embryos cultured sequentially from days 1 to 3 followed by days 4 to 8 in the different combinations of cell-conditioned and non-cell-conditioned media. Data are presented as least squares means (LSM) ± s.e. of the LSM (s.e.m .). Number (*n*) of observations for each treatment are presented in Supplementary Table 2.

**Figure 4:**
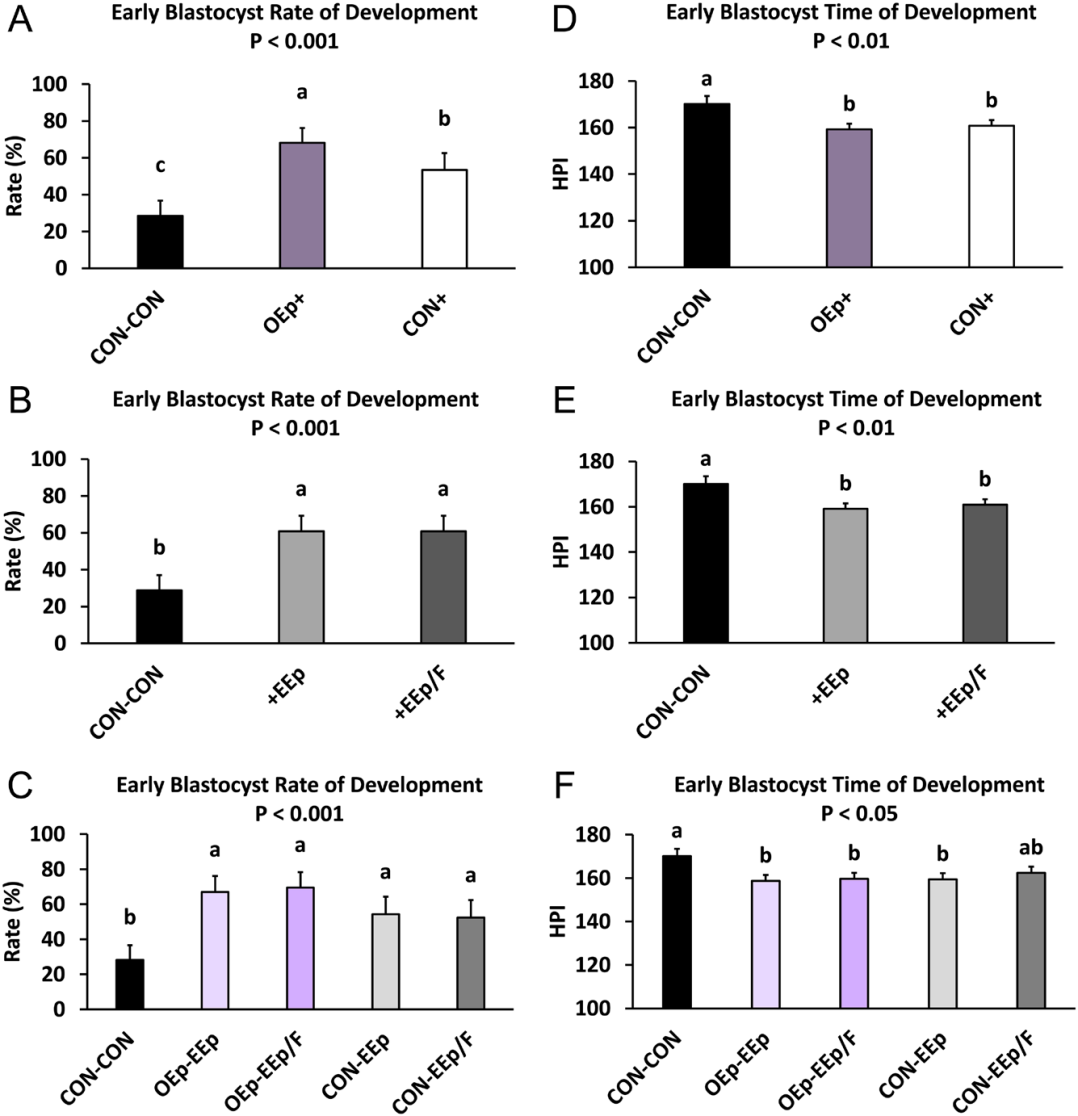
Percentage of early blastocyst development and time of development during sequential embryo culture in bovine reproductive cell-conditioned potassium simplex optimized medium (KSOM). (A and D; statistical analysis 1; SA1) Percentage of early blastocyst development and time of development (hours post insemination; (HPI)) between days 4 and 8 for embryos sequentially cultured in non-cell-conditioned KSOM from days 1 to 3 followed by days 4 to 8 (CON-CON) and embryos cultured in oviduct epithelial cell- conditioned KSOM or CON from days 1 to 3 (OEp+ or CON+, respectively) followed by culture in endometrial epithelial (EEp) and endometrial epithelial and fibroblast cell (EEp/F; coculture) conditioned KSOM (EEp and EEp/F data combined) from days 4 to 8. (B and E; SA2) Percentage of early blastocyst development and time of development between days 4 and 8 for embryos sequentially cultured in CON-CON and embryos cultured in EEp or EEp/F cell-conditioned KSOM from days 4 to 8 (+EEp or +EEp/F, respectively) after initial culture in oviduct epithelial cell-conditioned KSOM (OEp) and CON (data combined) from days 1 to 3. (C and F; SA3) Percentage of early blastocyst development and time of development (HPI) between days 4 and 8 for embryos cultured sequentially from days 1 to 3 followed by days 4–8 in the different combinations of cell conditioned and non-cell-conditioned media. Data are presented as least squares means (LSM) ± s.e. of the LSM (s.e.m.). Number (*n*) of observations for each treatment are presented in Supplementary Table 2.

Regarding oviduct cell-conditioned media (SA1), culturing IVP bovine embryos in OEp-conditioned KSOM from days 1 to 3 increased the percentage of compact morula and early blastocysts formed (SA1; treatment *P* < 0.01; Figs. 3, 4 and Supplementary Table 3) and reduced the time to compact morula and early blastocyst formation between days 4 and 8 (SA1; treatment *P* < 0.01; Figs. 3, 4 and Supplementary Table 3). Compared to CON-CON (*P* < 0.001) or CON+ (*P* ≤ 0.05), compact morula and early blastocyst development was greater when embryos were cultured in OEp+ from days 1 to 3 (Figs. 3, 4 and Supplementary Table 3). Further, compact morula and early blastocyst development was greater for CON+ when compared to CON-CON (*P* ≤ 0.05) indicating an effect of the endometrial cells on compact morula and blastocyst formation. Regarding time to morula formation, compared to CON-CON (*P* < 0.001) or CON+ (*P* < 0.05), culturing embryos in OEp+ medium from days 1 to 3 decreased the time to morula formation (Fig. 3 and Supplementary Table 3). The time to morula formation also decreased when embryos were cultured in CON+ compared to CON-CON (*P* < 0.05; Fig. 3 and Supplementary Table 3). Although no differences were found between OEp or CON+, compared to the CON-CON treatment, the time to early blastocysts formation was less for embryos cultured in OEp+ (*P* < 0.001) and CON+ (*P* < 0.01) (Fig. 4 and Supplementary Table 3).

Regarding endometrial cell-conditioned media (SA2), culturing embryos in the endometrial cell-conditioned KSOM from days 4 to 8 increased the percentage of compact morula and early blastocysts formed (SA2; treatment *P* < 0.05; Figs. 3, 4 and Supplementary Table 3) and reduced the time to compact morula and early blastocyst formation (SA2; treatment *P* < 0.01; Figs. 3, 4 and Supplementary Table 3). Compared to CON-CON, embryos cultured in +EEp (*P* < 0.01) and +EEp/F (*P* < 0.01) had a greater percentage of compact morula and early blastocysts (Figs. 3, 4, and Supplementary Table 3). In addition, compared to CON-CON, embryos cultured in +EEp (*P* < 0.01) and +EEp/F (*P* < 0.01) had a reduced time to compact morula and early blastocyst formation (Figs. 3, 4, and Supplementary Table 3). No differences were found in percent or time of formation of compact morula or early blastocysts between +EEp and +EEp/F treatments.

Regarding the sequential cell-conditioned media culture (SA3), sequential culture of embryos in OEp and endometrial cell-conditioned KSOM from days 1 to 8 increased the percentage of compact morula and early blastocysts that developed (SA3; treatment *P* < 0.05; Figs. 3, 4and Supplementary Table 3) and reduced the time to compact morula and early blastocyst formation (treatment *P* < 0.05; Figs. 3, 4 and Supplementary Table 3). Numerically, all treatments involving a cell-conditioned KSOM resulted in a greater percentage of compact morula compared to CON-CON although the OEp-EEp (*P* = 0.05) and OEp-EEp/F (*P* < 0.05) treatments had significantly greater compact morula (Fig. 3 and Supplementary Table 3). Regarding early blastocysts, compared to CON-CON, culturing embryos in OEp-EEp (*P* < 0.001), OEp-EEp/F (*P* < 0.001), CON-EEp (*P* < 0.05), or CON-EEp/F (*P* < 0.05) resulted in a greater percentage of early blastocysts. Further, compared to CON-CON, embryos cultured in OEp-EEp (*P* < 0.05), OEp-EEp/F (*P* < 0.05), or CON-EEp (*P* < 0.05) developed into compact morula and early blastocysts more quickly (Figs. 3, 4 and Supplementary Table 3).

### Effect of cell-conditioned medium on normal and expanded blastocyst formation

**Figure 5:**
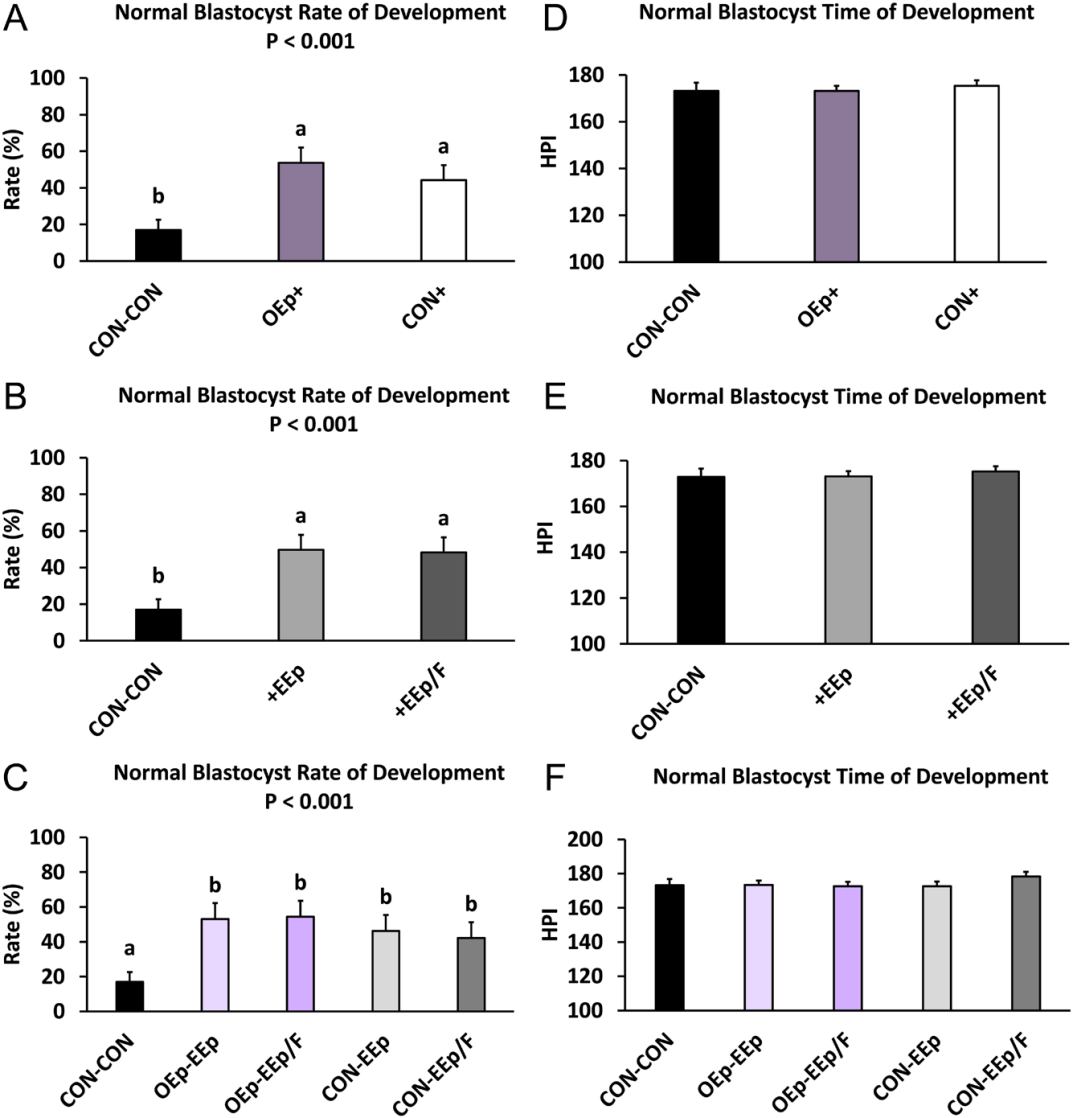
Percentage of normal blastocyst development and time of development during sequential embryo culture in bovine reproductive cell-conditioned potassium simplex optimized medium (KSOM). (A and D; statistical analysis 1; SA1) Percentage of normal blastocyst development and time of development (hours post insemination (HPI)) between days 4 and 8 for embryos sequentially cultured in non-cell-conditioned KSOM from days 1 to 3 followed by days 4–8 (CON-CON) and embryos cultured in oviduct epithelial cell-conditioned KSOM or CON from days 1 to 3 (OEp+ or CON+, respectively) followed by culture in endometrial epithelial (EEp) and endometrial epithelial and fibroblast cell (EEp/F; coculture) conditioned KSOM (EEp and EEp/F data combined) from days 4 to 8. (B and E; SA2) Percentage of normal blastocyst development and time of development (HPI) between days 4 and 8 for embryos sequentially cultured in CON-CON and embryos cultured in EEp or EEp/F cell-conditioned KSOM from days 4 to 8 (+EEp or +EEp/F, respectively) after initial culture in oviduct epithelial cell-conditioned KSOM (OEp) and CON (data combined) from days 1 to 3. (C and F; SA3) Percentage of normal blastocyst development and time of development (HPI) between days 4 and 8 for embryos cultured sequentially from days 1 to 3 followed by days 4–8 in the different combinations of cell-conditioned and nonconditioned media. Data are presented as least squares means (LSM) ± s.e. of the LSM (s.e.m.). Number (*n*) of observations for each treatment are presented in Supplementary Table 2.

**Figure 6:**
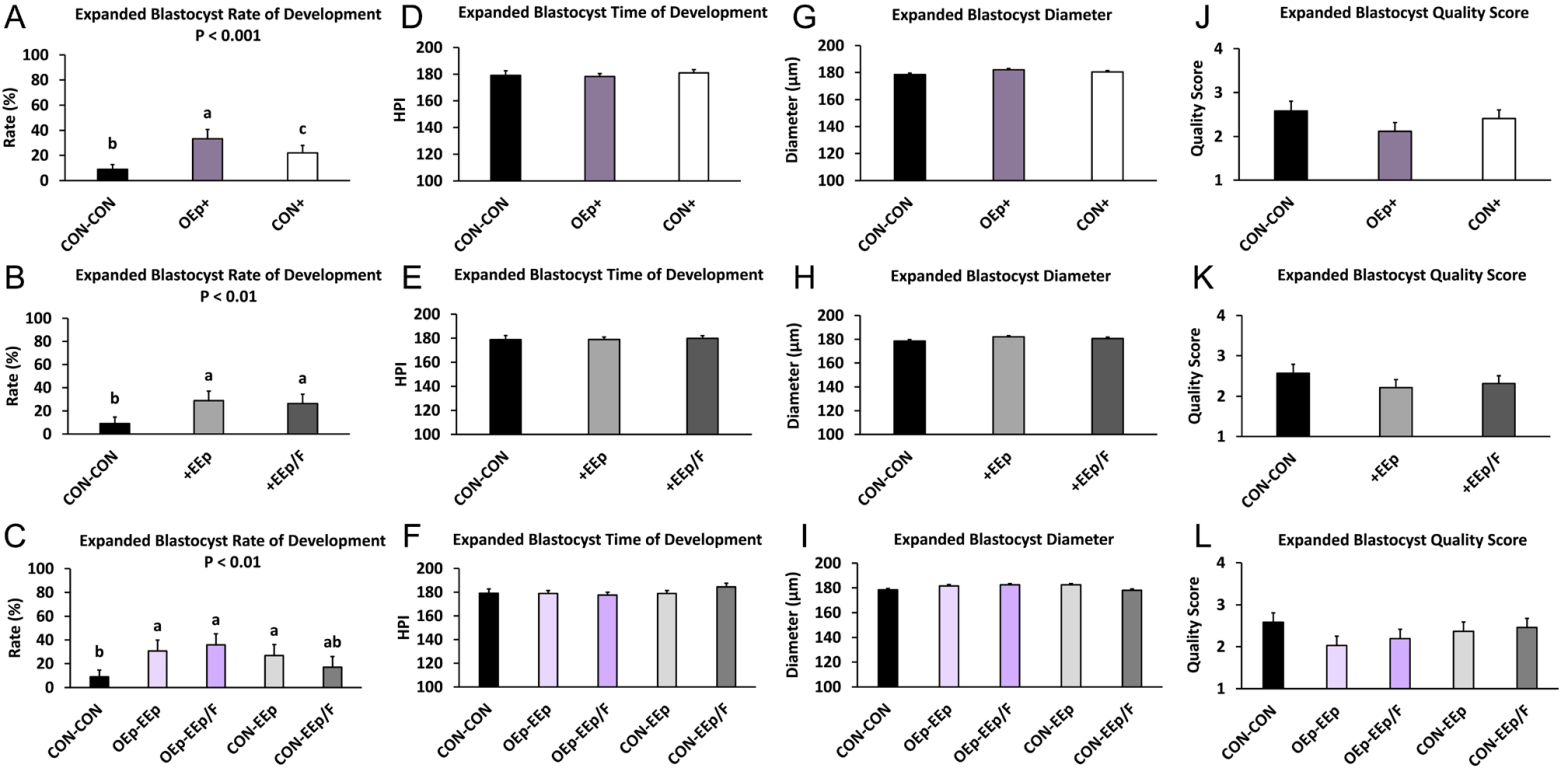
Percentage of expanded blastocyst development and time of development during sequential embryo culture in bovine reproductive cell-conditioned potassium simplex optimized medium (KSOM) as well as day 8 expanded blastocyst diameter and quality score (1–4, excellent/good to degenerating/dead, respectively). (A, D, G, and J; statistical analysis 1; SA1) Percentage of expanded blastocyst development and time of development (hours post insemination; (HPI)) between days 4 and 8 as well as day 8 expanded blastocyst diameter and quality score for embryos sequentially cultured in non-cell-conditioned KSOM from days 1–3 followed by days 4–8 (CON-CON) and embryos cultured in oviduct epithelial cell-conditioned KSOM or CON from days 1–3 (OEp+ or CON+, respectively) followed by culture in endometrial epithelial (EEp) and endometrial epithelial and fibroblast cell (EEp/F; coculture) conditioned KSOM (EEp and EEp/F data combined) from days 4 to 8. (B, E, H, and K; SA2) Percentage of expanded blastocyst development and time of development (HPI) between days 4 and 8 as well as day 8 expanded blastocyst diameter and quality score for embryos sequentially cultured in CON-CON and embryos cultured in EEp or EEp/F cell-conditioned KSOM from days 4 to 8 (+EEp or +EEp/F, respectively) after initial culture in oviduct epithelial cell-conditioned KSOM (OEp) and CON (data combined) from days 1 to 3. (C, F, I, and L; SA3) Percentage of expanded blastocyst development and time of development (HPI) between Days 4 and 8 as well as day 8 expanded blastocyst diameter and quality score for embryos cultured sequentially from days 1 to 3 followed by days 4 to 8 in different combinations of cell conditioned and non-cell-conditioned media. Data are presented as least squares means (LSM) ± s.e. of the LSM (s.e.m.). Number (*n*) of observations for each treatment are presented in Supplementary Table 2.

Regarding oviduct cell-conditioned media (SA1), normal and expanded blastocyst development were similar during the study. Culturing embryos from days 1 to 3 in OEp-conditioned KSOM increased the percentage of normal and expanded blastocysts between days 4 and 8 (SA1; treatment *P* < 0.001; Figs. 5, 6 and Supplementary Table 3) but did not affect the time to normal or expanded blastocyst formation. Compared to CON-CON, culturing embryos in OEp+ or CON+ resulted in a greater percentage of normal (OEp+, *P* < 0.001; CON+, *P* < 0.001) and expanded (OEp+, *P* = 0.001; CON+, *P* < 0.05) blastocysts (Figs. 5, 6 and Supplementary Table 3). There were no differences between OEp+ and CON+ in terms of percentage of normal blastocysts; however, treating embryos with OEp+ resulted in a greater percentage of expanded blastocyst compared to CON+ (*P* < 0.05; Figs. 5, 6 and Supplementary Table 3).

Regarding endometrial cell-conditioned media (SA2), culturing embryos in endometrial cell-conditioned KSOM from day 4 to 8 resulted in a greater percent of normal (SA2; treatment *P* < 0.001; Figs. 5, 6 and Supplementary Table 3) and expanded blastocysts (SA2; treatment *P* < 0.01; Figs. 5, 6 and Supplementary Table 3) but did not affect the time to blastocyst formation. Specifically, culturing embryos in +EEp or +EEp/F resulted in a greater percentage of normal (+EEp, *P* < 0.001; +EEp/F, *P* < 0.001) and expanded (+EEp, *P* < 0.001; +EEp/F, *P* < 0.01) blastocysts when compared to CON-CON (Figs. 5, 6 and Supplementary Table 3). There were no differences in the percentage of normal and expanded blastocysts that formed between embryos cultured in the +EEp- and +EEp/F-conditioned media and the time to blastocyst formation was similar across all treatments (Figs. 5, 6 and Supplementary Table 3).

Regarding the sequential cell-conditioned media culture (SA3), sequential culture of embryos in OEp followed by endometrial cell-conditioned media increased normal (SA3; Treatment *P* < 0.001; Figs. 5, 6 and Supplementary Table 3) and expanded (SA3; Treatment *P* < 0.01; Figs. 5, 6 and Supplementary Table 3) blastocyst development but did not affect the time to normal or expanded blastocyst development. Specifically, compared to CON-CON, embryos cultured in OEp-EEp (*P* < 0.001), OEp-EEp/F (*P* < 0.001), CON-EEp (*P* < 0.01), or CON-EEp/F (*P* < 0.01) had a greater percentage of normal blastocysts (Figs. 5, 6 and Supplementary Table 3) although no differences were detected between cell-conditioned media. Compared to CON-CON, embryos cultured in OEp-EEp (*P* < 0.01), OEp-EEp/F (*P* < 0.01), or CON-EEp (*P* < 0.05) media also had a greater percentage of expanded blastocysts (Figs. 5, 6 and Supplementary Table 3). Like normal blastocysts, no differences in the percentage of expanded blastocysts were detected between the cell-conditioned media. Further, there was no difference between treatment regarding time to normal or expanded blastocyst formation (Figs. 5, 6 and Supplementary Table 3).

### Effect of cell-conditioned medium on expanded blastocyst grade, diameter, and hatching

Blastocysts that reached the expanded blastocyst stage were evaluated for quality grade and diameter on day 8 (192 HPI). No statistical differences were found between treatments regarding quality score; however, embryos cultured in CON-CON consistently had a higher average grade (reduced quality score) compared to embryos cultured in cell-conditioned media (SA1–3; Fig. 6 and Supplementary Table 3). No statistical differences or numerical trend in the data between treatments were found for blastocyst diameter (Fig. 6 and Supplementary Table 3). Blastocysts from the CON-CON or CON-EEp/F treatments did not hatch while in culture and only 2.6% (2/77) of CON-EEp, 6.5% (5/77) of OEp-EEp, and 3.9% (3/77) of OEp-EEp/F embryos hatched at an average of 185, 192, and 190 HPI, respectively.

### Effect of cell-conditioned medium on day 8 blastocyst cell number

**Figure 7:**
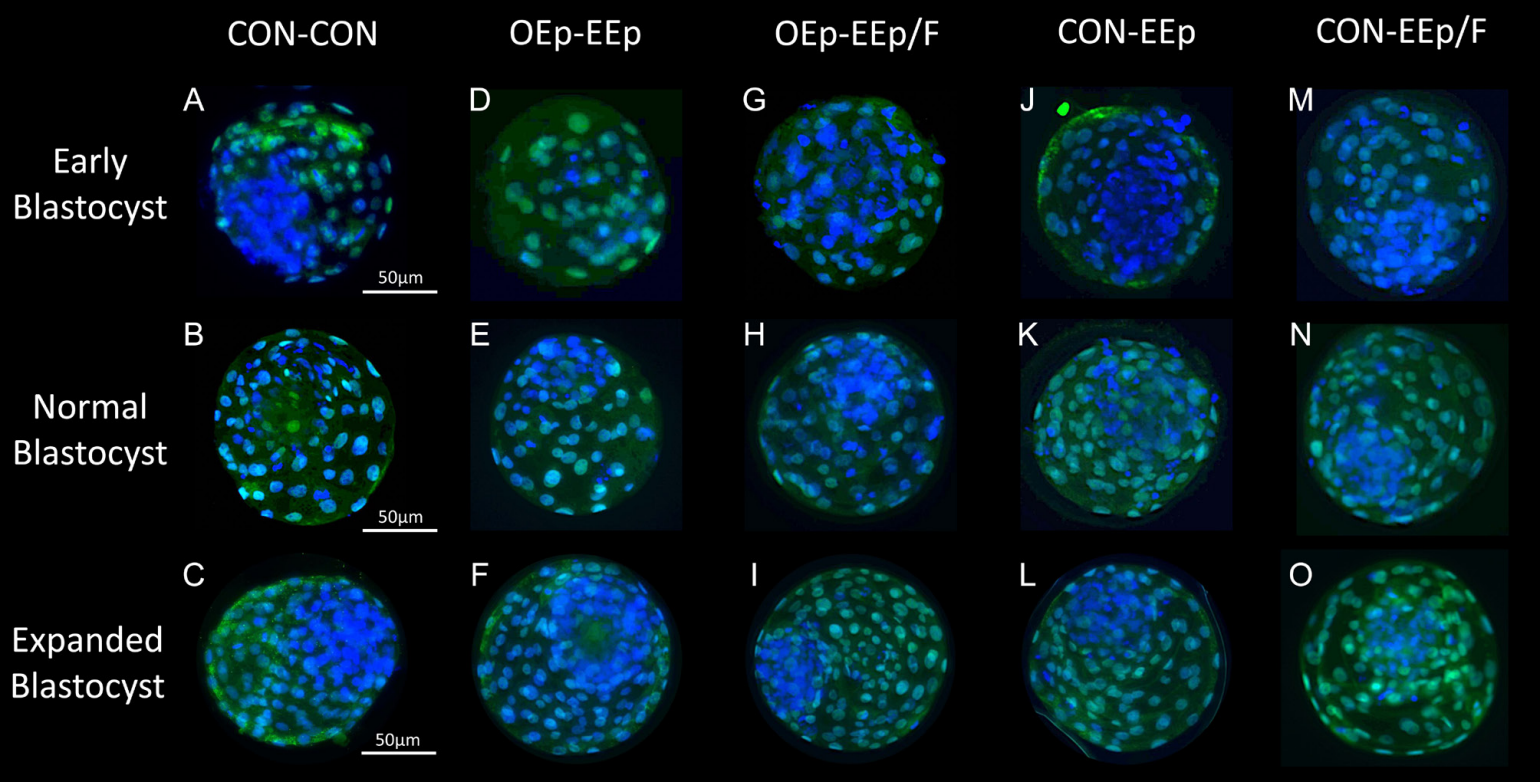
Immunohistochemistry (IHC) images of early (A–M), normal (B–N), and expanded (C–O) day 8 bovine blastocysts after sequential culture in non-cell-conditioned (CON) potassium simplex optimized medium (KSOM) or oviduct epithelial cell (OEp)-conditioned KSOM followed by either CON or endometrial epithelial cell (EEp) or endometrial epithelial and fibroblast cell (EEp/F) conditioned KSOM. During IHC, blastocyst TE caudal-type homeobox 2 (CDX-2) was stained using an indirect immunostaining technique involving a green, fluorescent labeled antibody. Blastocysts were then mounted in Fluoromount G containing 4’,6-diamidino-2-phenylindole (DAPI; blue) to stain all blastomere nuclei. Images of the blastocysts were taken using a camera and microscope equipped for fluorescent green and blue detection and the number of green labeled TE cells were counted and subtracted from the total number of cells (blue) to determine the number of ICM cells. The panel above presents merged fluorescent images of select blastocysts. Overall, there was no effect of treatment on the number or percentage of cells or ratio of TE to ICM cells. Bar = 50 µm. Early, normal, and expanded blastocyst cell number data is presented in Supplementary Tables 4, 5, and 6.

Early, normal and expanded blastocysts with a quality score of 3 or greater at 192 HPI were stained for CDX2 and cell nuclei to measure the total, ICM and TE cell numbers as well as the ICM to TE ratio. No statistical differences between the treatments across the different blastocyst stages were found (Supplementary Tables 4, 5, and 6). Representative images of the blastocysts from each treatment are presented in Fig. 7.

## Discussion

The aim of this study was to test if bovine oviduct and endometrial cells could be used to improve IVP bovine embryo development by conditioning KSOM, a standard embryo culture medium. Overall, major findings from this study include: i) culturing IVP bovine embryos with oviduct or endometrial cell-conditioned KSOM increases IVP embryo structure formation and rate of structure formation and ii) culturing IVP bovine embryos sequentially in oviduct followed by endometrial cell-conditioned KSOM has the greatest impact on embryo developmental kinetics, specifically, increased compact morula and blastocyst formation as well as rate of formation.

In cattle, fertilization occurs within the oviduct near the ampulla–isthmus junction. Over the course of the next four days, the embryo will migrate through the isthmus lumen toward the uterine lumen while undergoing initial cell divisions. The isthmus lumen is surrounded by the endosalpinx which consists of a highly secretory epithelium supported by underlying fibroblast cells. For this reason, OEp have been used extensively with the aim of improving IVP embryo culture conditions. Indeed, early coculture systems involving IVP cattle embryos and OEp, or cell supernatants allowed investigators to overcome the embryo 8–16-cell ‘developmental block’ (Eyestone & First 1989, Marquant-Le Guienne *et al.* 1989, Mermillod *et al.* 1993). Since then, studies involving culture of IVP bovine embryos in ewe oviducts *in vivo* or coculture with oviduct fluid, OEp, or OEp-conditioned media have reported greater embryo interferon tau production, blastocyst formation, and/or embryo quality following cryopreservation (Lonergan *et al.* 2003, Cordova *et al.* 2014, Lopera-Vasquez *et al.* 2017, Talukder *et al.* 2018). Studies investigating the effect of OEp specifically on advanced PZ cleavage to the two-cell stage have been less conclusive. Cordova *et al.* (2014) found that culturing PZ with OEp during initial development (days 1–4), when embryos would normally be within the oviduct, resulted in greater PZ cleavage and blastocyst formation and concluded that OEp accelerated embryo developmental kinetics. In this study, we did not find an effect of OEp-conditioned media on PZ cleavage. There are several differences between our study and that of Cordova *et al.* (2014), including culture media; however, it is possible we missed any advantage that OEp had on PZ cleavage as we assessed this manually at 64 HPI, whereas Cordova *et al.* (2014) assessed cleavage at approximately 24 HPI. Embryo culture with the conditioned media or OEp was initiated at 16 and 20 HPI, respectively, during the two studies. Within the oviduct, cleavage typically occurs in cattle between 24 and 28 HPI, although the event is typically delayed for IVP bovine embryos occurring approximately 26 and 32 HPI (Lonergan *et al.* 1999, Holm *et al.* 2002). It is also possible that the secretory capacity of the cells was not optimally synchronized with that of the developmental stage of the embryo. In this study, the OEp and EEp/F cells were isolated during the early to mid-luteal phase (diestrus) of the estrous cycle and not specifically during metaestrus when the maternal endocrine profile would be considerably different. Similar to a study by Hamdi *et al.* (2018), we also did not detect an effect of OEp-conditioned media on the number of 8–16-cell embryos that developed. However, IVP embryo developmental kinetics was accelerated during our study because culturing IVP bovine embryos in OEp-conditioned media from days 1 to 3 resulted in a greater number of morula and blastocysts and reduced time to morula and blastocyst formation between days 4 and 8 of development, phenomena we were able to detect using an embryo time-lapse incubator.

Oviduct epithelial cells likely secrete embryotropic substances and/or modified key energy substrates within the KSOM that promote advanced morula and blastocyst formation. Prior to morula compaction, bovine oocytes and embryos rely heavily on pyruvate and lactate provided by the oviduct and/or cumulus cells for energy (Gardner & Harvey 2015). Near the 16-cell stage, coinciding with energy demanding processes such as compaction, embryonic genome activation, and subsequent blastocyst formation, bovine embryos increase aerobic respiration and aerobic glycolysis (Gardner & Harvey 2015). In line with this, it was found that bovine OEp monolayers lowered tissue culture media 199 (TCM-199) glucose concentrations and increased pyruvate and lactate levels (Edwards *et al.* 1997). Additionally, nonessential amino acids, such as glutamine/glutamic acid, and essential amino acids levels were increased while several others were decreased when compared to non-cell-conditioned TCM-199 (Rispoli *et al.* 2011). Glutamine also plays an important role in embryo energy production prior to morula compaction (Gardner & Harvey 2015, Rieger *et al.* 1992). Potassium simplex optimized medium is formulated with pyruvic acid, lactic acid, glucose, glutamine, and OEp may modify concentrations of these molecules to better suit pre-compaction embryo requirements for subsequent development. Bovine OEp were also found to reduce media oxygen concentrations which can induce bovine blastocyst expression of glucose transporter 1 (*GLUT1*), also known as solute carrier *2A1* (Harvey *et al.* 2007, Cordova *et al.* 2014, Gardner & Harvey 2015). Thus, culturing bovine embryos in media conditioned by OEp may have reduced media oxygen and boosted post-compaction embryo expression of GLUT1 and glucose uptake for accelerated morula and blastocyst development.

Various micro- and macro-molecules including cytokines, growth factors, and extracellular vesicles are secreted by the oviduct and endometrial epithelium and are hypothesized to support embryonic development (Lee & Yeung 2006, Wang *et al.* 2009, Brooks *et al.* 2016, Burns *et al.* 2018, Leal *et al.* 2022, Mathew *et al.* 2022, Pushpakumara *et al.* 2002). These factors may have been secreted by the OEp or EEp in this study resulting in accelerated compact morula and blastocyst formation. Specifically, in cattle, insulin-like growth factor-I (IGF-I) is secreted by bovine OEp and IGF-I receptors (IGF-IR) are expressed by the early bovine embryo (Pushpakumara *et al.* 2002, Wang *et al.* 2009), possibly contributing to blastocyst formation (Matsui *et al.* 1997, Wang *et al.* 2012, Kaya *et al.* 2017). During a study by Leal *et al.* (2022), investigators cultured IVP bovine PZ with extracellular vesicles isolated from mid-luteal phase bovine oviducts. Although extracellular vesicles did not impact blastocyst formation, embryos cultured with the vesicles had greater total cell numbers, altered gene expression, and improved survival rates following vitrification (Leal *et al.* 2022).

The endometrium is highly secretory and contributes endometrial secretions, collectively termed histotroph, that completely support embryonic development between days 4 and 20 of gestation in cattle (Mathew *et al.* 2022). The importance of endometrial secretions, particularly from the GE, in pre-implantation embryonic development has been elegantly shown in uterine gland knock-out studies in sheep as normal embryonic development including elongation does not occur in sheep lacking uterine glands (Gray *et al.* 2001). During early studies, bovine endometrial stromal fibroblast cell monolayers (i.e. EF) were tested for their capacity to improve *in vitro* cultured embryo development during coculture with some success, mostly related to blastocyst quality (Kuzan & Wright 1982, Voelkel *et al.* 1985). More recent studies involving bovine EEp have reported increased blastocyst formation when IVP bovine embryos are cocultured with EEp monolayers or cell-conditioned media (Sponchiado *et al.* 2020, Oliver *et al.* 2023). During a study by Lakshmi Devi *et al.* (2022), coculture of IVP buffalo (Bubaline) embryos with P4 and estradiol (E2)-stimulated EEp or cell-conditioned media improved blastocyst formation and hatching. Similarly, Sponchiado *et al.* (2020) reported a greater number of normal, expanded, and hatched IVP bovine blastocysts when embryos were cocultured with bovine EEp in SOF (5% FBS). Culture of embryos in cell-conditioned media also increased normal blastocyst formation but had no effect on the number of expanded or hatched blastocysts (Sponchiado *et al.* 2020).

**Figure 8:**
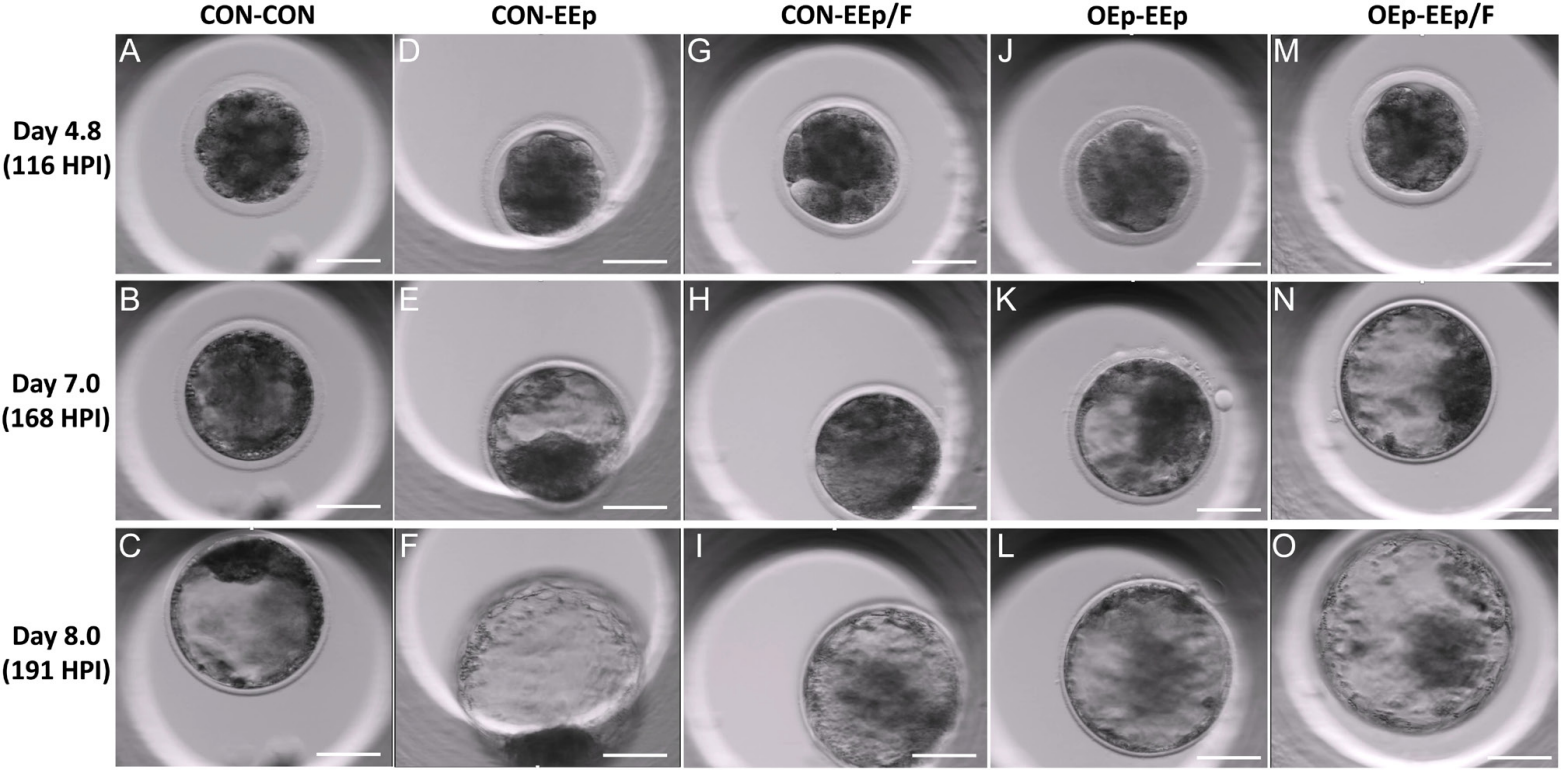
MIRI TL6 images of IVP bovine embryos on days 4.8 (116 post insemination (HPI)), 7 (168 HPI) and 8 (191 HPI) of development after culture in bovine reproductive cell or non-cell-conditioned potassium simplex optimized media (KSOM). (A–C) An embryo cultured from days 1 to 3 (CON) followed by days 4–8 (CON) in non-cell-conditioned KSOM (CON-CON). (D–F) An embryo cultured in non-cell-conditioned KSOM from days 1–3 followed by endometrial epithelial cell (EEp) conditioned KSOM from days 4 to 8 of development (CON-EEp). (G–I) An embryo cultured in non-cell-conditioned KSOM from days 1 to 3 followed by endometrial epithelial and fibroblast cell (EEp/F) conditioned KSOM from days 4 to 8 of development (CON-EEp/F). (J–L) An embryo cultured in oviduct epithelial cell (OEp)-conditioned KSOM from days 1 to 3 followed by EEp-conditioned KSOM from days 4 to 8 of development (OEp-EEp). (M-O) An embryo cultured in OEp-conditioned KSOM from days 1 to 3 followed by EEp/F-conditioned KSOM from days 4 to 8 of development. Bar = 50 µm.

During a recent study, we also detected an increase in IVP bovine blastocyst formation when embryos were cultured in endometrial cell-conditioned media (Oliver *et al.* 2023). The conditioned media were prepared by culturing primary bovine EEp or EF for 12 h in a standard cell culture media (Roswell Park Memorial Institute; RPMI) containing 5% FBS and 15 ng of P4. Culturing embryos in the EEp, EF or a combined (1:1) cell-conditioned media after day 4 of development increased blastocyst formation over nonconditioned media (control) by day 7 (Oliver *et al.* 2023). Further, the number of blastocysts within the cell-conditioned media was numerically greater than that of IVP embryos cultured in KSOM. By day 8, however, only the EEp cell-conditioned media had a greater number of blastocysts compared to Control and the number of blastocysts within KSOM numerically surpassed that of all other treatments (Oliver *et al.* 2023). Like our previous study, culturing IVP bovine embryos from days 4 to 8 in EEp-conditioned media without prior culture in OEp-conditioned media increased the number of blastocysts compared to the control (CON-CON). Although no differences in development were detected between the CON-EEp and CON-EEp/F-conditioned media treatments, collectively, embryo developmental kinetics and morula and blastocyst formation were greatest when embryos were cultured in OEp followed by EEp (OEp-EEp) or EEp/F (OEp-EEp/F) conditioned media (Fig. 8). Like OEp, EEp may have modified KSOM via cellular secretion of embryotropic molecules and/or modification of key energy substrates. Unlike our previous study, however, we did not supplement KSOM with P4, indicating benefits provided by the reproductive cells on embryonic development were independent of this major ovarian steroid.

*In vivo*, EEp and EF maintain important cell-to-cell interactions and paracrine communication to support endometrial remodeling, cell differentiation, and optimal endometrial secretions for pregnancy success (Chaney *et al.* 2021). Chen *et al.* (2013) evaluated the effects of coculturing human EEp and EF in a 2D cell culture system and compared expression profiles to that of monocultured cells (Chen *et al.* 2013). Compared to monocultured cells, cocultured EEp and EF maintained activities more like *in vivo* phenotypes and cocultured cells expressed or secreted factors related to structural integrity and immunity including several chemokines and interleukins (Chen *et al.* 2013). For these reasons, we cultured EEp alone or with EF (EEp/F) and tested the effect of cell coculture on media conditioning and embryonic development. Overall, we did not detect a benefit of EEp/F coculture over EEp alone in this study; however, it is clear that EEp optimized media conditions for advanced embryonic development, particularly for embryos previously cultured in OEp-conditioned media. It is possible that OEp secretions in the conditioned media induced expression of endometrial histotroph molecule receptors on the embryo, which could have been stimulated by EEp secretions within the EEp or EEp/F-conditioned media between days 4 and 8 of development. Theoretically, sequential culture of bovine embryos in the presence of OEp followed by EEp or EEp/F secretions would more closely mimic the embryo environment during *in vivo* development (i.e., migration of the embryo from the oviduct to the uterus).

We did not detect a statistical effect of cell-conditioned media on blastocyst diameter, hatching, or quality; however, embryos cultured in any combination of cell-conditioned media numerically had better quality scores compared to embryos cultured in non-cell-conditioned media (Fig. 6). Embryo hatching, which was minimal, only occurred within CON-EEp, OEp-EEp, or OEp-EEp/F treatments. Using a similar staining technique as used in this study, Sponchiado *et al.* (2020) reported a greater number of total cells but no difference in the number of TE and ICM cells within day 7.5 bovine embryos cocultured with EEp compared to control. We did not detect an effect of cell-conditioned media on day 8 embryo cell numbers in this study. If advanced embryo developmental kinetics and quality in cell-conditioned media are not related to blastomere proliferation, it is possible that oviduct and endometrial cells support compaction and blastocoel formation through advanced blastomere plasma membrane integrity, tight junction formation, and/or expression of carriers and channels such as the sodium–potassium ATPase pump or aquaporins (Houghton *et al.* 2003, Petano-Duque *et al.* 2022).

## Conclusion

Culture of IVP bovine embryos in endometrial or oviduct cell conditioned media or sequentially in oviduct followed by endometrial cell conditioned media improved IVP bovine embryo developmental kinetics, survival, and quality *in vitro*. The benefit of the reproductive cells may be provided through cell modification of the media and availability of key energy substrates and/or secretion of embryotropic molecules. Overall, compared to non-cell-conditioned media, embryo sequential culture in oviduct followed by endometrial cell (EEp or EEp/F) conditioned media had the greatest impact on advanced embryonic development possibly because it more closely mimics the *in vivo* embryo environment. Important questions remain, however, such as which embryotropic molecules provide these effects and if the cell conditioned media support advanced embryonic survival and pregnancy success post embryo transfer. Answers to these questions could lead to technologies that improve IVP embryo survival and reduce pregnancy failure associated with IVP bovine embryos.

## Supplementary Materials

Supplementary Table 1. Number (n) of observations related to embryo structure formation rate of development (percentage) and time of embryo structure formation [hours post-insemination (HPI)] as well as expanded blastocyst (ExB) quality grade score and diameter for statistical analysis (SA) 1, 2 and 3 of the study.

Supplementary Table 2. A summary of binomial data and continuous or categorical data found to have a normal distribution after a Shapiro-Wilk test (> 0.90; normal distribution). All data were analyzed using a Proc GLIMMIX in SAS, where the distribution was designated as binomial or normal.

Supplementary Table 3. Data related to embryo structure formation rate of development (percentage) and time of embryo structure formation [hours post-insemination (HPI)] as well as expanded blastocyst (ExB) quality grade score and diameter for statistical analysis (SA) 1, 2 and 3 of the study.

Supplementary Table 4. Data related to early blastocyst (EB) trophectoderm (TE) and inner cell mass (ICM) cell number and percentage as well as the total cell number and the ICM:TE ratio for statistical analysis (SA) 1, 2 and 3 of the study.

Supplementary Table 5. Data related to normal blastocyst (NB) trophectoderm (TE) and inner cell mass (ICM) cell number and percentage as well as the total cell number and the ICM:TE ratio for statistical analysis (SA) 1, 2 and 3 of the study.

Supplementary Table 6. Data related to expanded blastocyst (ExB) trophectoderm (TE) and inner cell mass (ICM) cell number and percentage as well as the total cell number and the ICM:TE ratio for statistical analysis (SA) 1, 2 and 3 of the study.

## Declaration of interest

The authors declare that there is no conflict of interest that could be perceived as prejudicing the impartiality of the study reported.

## Funding

This project was supported by Agriculture and Food Research Initiative Competitive Grant no. 2020-67015-31617 from the USDA National Institute of Food and Agriculture, the University of Tennessee Graduate School and AgResearch, the Department of Animal Science and the USDA National Institute of Food and Agriculture, Hatch Project No. 1022068.

## Author contribution statement

LKS: *in vitro* embryo production, reproductive cell culture, data collection, and manuscript preparation; KDP: *in vitro* embryo production and manuscript preparation; JLE: *in vitro* embryo production and manuscript preparation; RRP: *in vitro* embryo production and manuscript preparation; DJM: experimental design, acquisition of funding, and manuscript preparation.
