# Supplementary material for: Oviduct and endometrial epithelium improve in vitro produced bovine embryo developmental kinetics
Source: Reproduction. 2024 Apr 17;167(5):e240008. doi: 10.1530/REP-24-0008 (PMC11056959; doi:10.1530/REP-24-0008)
Supplement: Supplementary Table 1. Number (n) of observations related to embryo structure formation rate of development (percentage) and time of embryo structure formation [hours post-insemination (HPI)] as well as expanded blastocyst (ExB) quality grade score and diameter for statistical analysis (SA) 1, 2 and [file supplementary_table_1.pdf]

Supplementary Table 1. Number (n) of observations related to embryo structure formation rate of development (percentage) and time of embryo structure formation [hours post-insemination (HPI)] as well as expanded blastocyst (ExB) quality grade score and diameter for statistical analysis (SA) 1, 2 and 3 of the study.

SA	TRT	CM %	CM (HPI)	EB %	EB (HPI)	NB %	NB (HPI)	ExB %	ExB (HPI)	ExB Grade	ExB Diameter
SA1	CON-CON	77	42	77	25	77	15	77	8	8	8
	OEp+	154	119	154	107	154	87	154	56	41	46
	CON+	154	103	154	87	154	73	154	38	29	32
SA2	CON-CON	77	42	77	25	77	15	77	8	8	8
	+EEp	154	111	154	97	154	81	154	49	39	40
	+EEp/F	154	112	154	97	154	79	154	45	31	38
SA3	CON-CON	77	42	77	25	77	15	77	8	8	8
	OEp-EEp	77	58	77	53	77	43	77	26	22	22
	OEp-EEp/F	77	61	77	54	77	44	77	30	19	24
	CON-EEp	77	53	77	44	77	38	77	23	17	18
	CON-EEp/F	77	51	77	43	77	35	77	15	12	14

TRT, treatment; CM, compact morula; EB, early blastocyst; NB, new blastocyst; ExB, Expanded blastocyst.
