# Supplementary material for: Oviduct and endometrial epithelium improve in vitro produced bovine embryo developmental kinetics
Source: Reproduction. 2024 Apr 17;167(5):e240008. doi: 10.1530/REP-24-0008 (PMC11056959; doi:10.1530/REP-24-0008)
Supplement: Supplementary Table 2. A summary of binomial data and continuous or categorical data found to have a normal distribution after a Shapiro-Wilk test (> 0.90; normal distribution). All data were analyzed using a Proc GLIMMIX in SAS, where the distribution was designated as binomial or normal. [file supplementary_table_2.pdf]

Supplementary Table 2. A summary of binomial data and continuous or categorical data found to have a normal distribution after a Shapiro-Wilk test (> 0.90; normal distribution). All data were analyzed using a Proc GLIMMIX in SAS, where the distribution was designated as binomial or normal.

Binomial	Continuous/Categorical
CM %	PZ cleavage % (well average)
EB %	8 to 16 cell embryo % (well average)
NB %	CM (HPI)
ExB %	EB (HPI)
	NB (HPI)
	ExB (HPI)
	ExB grade
	ExB diameter

PZ, presumptive zygote; HPI, hours post-insemination; CM, compact morula; EB, early blastocyst; NB, new blastocyst; ExB, Expanded blastocyst.
