# Supplementary material for: Oviduct and endometrial epithelium improve in vitro produced bovine embryo developmental kinetics
Source: Reproduction. 2024 Apr 17;167(5):e240008. doi: 10.1530/REP-24-0008 (PMC11056959; doi:10.1530/REP-24-0008)
Supplement: Supplementary Table 3. Data related to embryo structure formation rate of development (percentage) and time of embryo structure formation [hours post-insemination (HPI)] as well as expanded blastocyst (ExB) quality grade score and diameter for statistical analysis (SA) 1, 2 and 3 of the study. [file supplementary_table_3.pdf]

Supplementary Table 3. Data related to embryo structure formation rate of development (percentage) and time of embryo structure formation [hours post-insemination (HPI)] as well as expanded blastocyst (ExB) quality grade score and diameter for statistical analysis (SA) 1, 2 and 3 of the study.

SA	TRT	CM %	CM (HPI)	EB %	EB (HPI)	NB %	NB (HPI)	ExB %	ExB (HPI)	ExB Grade	ExB Diameter
SA1	CON-CON	52.7 ± 8.3^{b}	142.8 ± 2.0^{a}	28.4 ± 8.4^{c}	170.1 ± 3.4^{a}	16.9 ± 5.8^{b}	173.2 ± 3.6	8.9 ± 3.9^{b}	177.3 ± 3.7	1.0 ± 0.3	178.8 ± 8.4
	OEp+	76.6 ± 5.5^{a}	134.2 ± 1.4^{c}	68.2 ± 8.0^{a}	159.3 ± 2.4^{b}	53.7 ± 8.2^{a}	173.1 ± 2.1	33.3 ± 7.4^{a}	178.2 ± 2.3	1.5 ± 0.2	184.2 ± 5.0
	CON+	66.3 ± 6.6^{c}	137.3 ± 1.5^{b}	53.5 ± 9.1^{b}	160.8 ± 2.5^{b}	44.2 ± 8.2^{a}	175.2 ± 2.2	22.0 ± 5.9^{c}	180.8 ± 2.5	1.5 ± 0.2	178.5 ± 5.5
	P-value	<0.01	<0.001	<0.001	<0.01	<0.001	NS	<0.001	NS	NS	NS
SA2	CON-CON	52.7 ± 8.2^{b}	142.5 ± 2.1^{a}	28.8 ± 8.2^{b}	170.0 ± 3.4^{a}	16.9 ± 5.7^{b}	172.9 ± 3.6	9.0 ± 3.8^{b}	177.1 ± 3.7	1.0 ± 0.3	178.9 ± 8.5
	+EEp	71.2 ± 6.1^{a}	135.5 ± 1.5^{b}	60.8 ± 8.4^{a}	159.1 ± 2.4^{b}	49.6 ± 8.2^{a}	173.0 ± 2.1	28.9 ± 6.8^{a}	178.8 ± 2.3	1.3 ± 0.2	180.8 ± 5.1
	+EEp/F	71.8 ± 6.0^{a}	136.0 ± 1.5^{b}	60.8 ± 8.4^{a}	160.9 ± 2.4^{b}	48.3 ± 8.2^{a}	175.2 ± 2.1	26.3 ± 6.4^{a}	179.8 ± 2.3	1.7 ± 0.2	183.2 ± 5.4
	P-value	<0.05	<0.01	<0.001	<0.01	<0.001	NS	<0.01	NS	NS	NS
SA3	CON-CON	52.6 ± 8.3^{b}	142.8 ± 2.0^{a}	28.2 ± 8.1^{b}	170.1 ± 3.4^{a}	16.9 ± 5.8^{b}	173.3 ± 3.7	8.9 ± 3.9^{b}	177.6 ± 3.6	1.0 ± 0.3	178.9 ± 8.5
	OEp-EEp	74.6 ± 6.8^{a}	134.4 ± 1.8^{b}	67.0 ± 9.2^{a}	158.7 ± 2.8^{b}	53.0 ± 9.2^{a}	173.3 ± 2.6	30.7 ± 8.0^{a}	178.9 ± 2.6	1.2 ± 0.2	182.4 ± 5.9
	OEp-EEp/F	78.7 ± 6.2^{a}	134.0 ± 1.8^{b}	69.6 ± 8.8^{a}	159.7 ± 2.6^{b}	54.4 ± 9.2^{a}	172.6 ± 2.6	35.9 ± 8.6^{a}	177.5 ± 2.5	1.8 ± 0.2	186.3 ± 6.3
	CON-EEp	67.7 ± 7.5^{ab}	136.2 ± 1.9^{b}	54.3 ± 10.0^{a}	159.7 ± 2.7^{b}	46.2 ± 9.2^{a}	172.4 ± 2.7	26.9 ± 7.5^{a}	178.6 ± 2.6	1.5 ± 0.2	178.6 ± 6.6
	CON-EEp/F	65.0 ± 7.8^{ab}	138.5 ± 1.9^{ab}	52.4 ± 10.1^{a}	162.4 ± 2.9^{ab}	42.2 ± 9.0^{a}	178.3 ± 2.8	17.0 ± 5.8^{ab}	184.3 ± 3.0	1.5 ± 0.3	178.4 ± 7.3
	P-value	<0.05	<0.01	<0.001	<0.05	<0.001	NS	<0.01	NS	NS	NS

TRT, treatment; CM, compact morula; EB, early blastocyst; NB, new blastocyst; ExB, Expanded blastocyst.
