# Supplementary material for: Oviduct and endometrial epithelium improve in vitro produced bovine embryo developmental kinetics
Source: Reproduction. 2024 Apr 17;167(5):e240008. doi: 10.1530/REP-24-0008 (PMC11056959; doi:10.1530/REP-24-0008)
Supplement: Supplementary Table 4. Data related to early blastocyst (EB) trophectoderm (TE) and inner cell mass (ICM) cell number and percentage as well as the total cell number and the ICM:TE ratio for statistical analysis (SA) 1, 2 and 3 of the study. [file supplementary_table_4.pdf]

Supplementary Table 4. Data related to early blastocyst (EB) trophectoderm (TE) and inner cell mass (ICM) cell number and percentage as well as the total cell number and the ICM:TE ratio for statistical analysis (SA) 1, 2 and 3 of the study.

SA	TRT	EB (n)	Total Cell Number	TE Cell Number	ICM Cell Number	TE Cell %	ICM Cell %	ICM:TE Ratio
SA1	CON-CON	6	96.1 ± 9.1	49.7 ± 4.8	45.3 ± 6.2	53.1 ± 4.1	47.0 ± 4.0	0.8 ± 0.1
	OEp+	11	87.1 ± 7.1	49.4 ± 3.7	37.7 ± 5.2	58.8 ± 3.5	41.2 ± 3.5	0.7 ± 0.1
	CON+	8	77.4 ± 7.9	44.4 ± 4.1	32.6 ± 5.5	58.2 ± 3.7	41.8 ± 3.7	0.9 ± 0.1
	P-value	NA	NS	NS	NS	NS	NS	NS
SA2	CON-CON	6	96.2 ± 9.3	50.0 ± 4.7	45.5 ± 6.2	53.0 ± 4.0	47.0 ± 4.0	0.9 ± 0.1
	+EEp	11	76.8 ± 7.2	44.7 ± 3.5	31.8 ± 5.1	59.6 ± 3.4	40.4 ± 3.4	0.7 ± 0.1
	+EEp/F	8	92.5 ± 8.8	51.0 ± 4.4	41.1 ± 6.0	56.8 ± 3.9	43.2 ± 3.9	0.7 ± 0.1
	P-value	NA	NS	NS	NS	NS	NS	NS
SA3	CON-CON	6	95.4 ± 9.8	50.1 ± 4.9	45.0 ± 6.5	52.8 ± 4.1	47.2 ± 4.1	0.9 ± 0.1
	OEp-EEp	8	84.0 ± 9.1	49.3 ± 4.5	34.5 ± 6.1	60.6 ± 3.8	39.4 ± 3.8	0.7 ± 0.1
	OEp-EEp/F	3	112.7 ± 15.4	56.6 ± 8.2	55.3 ± 9.7	51.9 ± 6.4	48.2 ± 6.4	1.0 ± 0.2
	CON-EEp	3	55.5 ± 12.9	31.9 ± 6.7	23.8 ± 8.2	57.3 ± 5.3	42.7 ± 5.3	0.7 ± 0.1
	CON-EEp/F	5	89.0 ± 10.2	51.5 ± 5.2	37.4 ± 6.7	58.5 ± 4.2	41.5 ± 4.2	0.7 ± 0.1
	P-value		NS	NS	NS	NS	NS	NS

TRT, treatment.
