# Supplementary material for: Oviduct and endometrial epithelium improve in vitro produced bovine embryo developmental kinetics
Source: Reproduction. 2024 Apr 17;167(5):e240008. doi: 10.1530/REP-24-0008 (PMC11056959; doi:10.1530/REP-24-0008)
Supplement: Supplementary Table 5. Data related to normal blastocyst (NB) trophectoderm (TE) and inner cell mass (ICM) cell number and percentage as well as the total cell number and the ICM:TE ratio for statistical analysis (SA) 1, 2 and 3 of the study. [file supplementary_table_5.pdf]

Supplementary Table 5. Data related to normal blastocyst (NB) trophectoderm (TE) and inner cell mass (ICM) cell number and percentage as well as the total cell number and the ICM:TE ratio for statistical analysis (SA) 1, 2 and 3 of the study.

SA	TRT	NB (n)	Total Cell Number	TE Cell Number	ICM Cell Number	TE Cell %	ICM Cell %	ICM:TE Ratio
SA1	CON-CON	3	101.3 ± 19.8	61.2 ± 12.2	39.4 ± 8.7	60.3 ± 3.3	39.7 ± 3.2	0.9 ± 0.1
	OEp+	12	105.9 ± 12.2	62.2 ± 7.5	44.2 ± 5.0	58.7 ± 1.7	42.1 ± 1.6	0.7 ± 0.1
	CON+	11	108.8 ± 12.7	59.1 ± 7.8	50.6 ± 5.2	54.5 ± 1.7	45.6 ± 1.7	0.7 ± 0.1
	P-value	NA	NS	NS	NS	NS	NS	NS
SA2	CON-CON	3	101.0 ± 19.3	60.7 ± 12.1	39.7 ± 8.3	60.3 ± 3.2	39.7 ± 3.1	0.7 ± 0.1
	+EEp	13	113.3 ± 11.4	62.0 ± 7.0	52.0 ± 4.7	54.5 ± 1.6	45.5 ± 1.5	0.9 ± 0.1
	+EEp/F	10	100.2 ± 12.3	59.8 ± 7.5	41.1 ± 5.1	59.5 ± 1.8	41.4 ± 1.7	0.7 ± 0.1
	P-value	NA	NS	NS	NS	NS	NS	NS
SA3	CON-CON	3	100.7 ± 20.1	61.0 ± 12.7	39.0 ± 8.5	60.3 ± 3.2	39.7 ± 3.1	0.7 ± 0.1
	OEp-EEp	6	110.6 ± 15.8	62.4 ± 10.0	48.6 ± 6.4	56.2 ± 2.3	43.8 ± 2.2	0.8 ± 0.1
	OEp-EEp/F	6	101.2 ± 15.2	61.9 ± 9.6	39.7 ± 6.2	61.2 ± 2.3	40.0 ± 2.2	0.7 ± 0.1
	CON-EEp	7	115.6 ± 14.6	61.0 ± 9.2	55.6 ± 5.9	53.0 ± 2.1	47.0 ± 2.1	0.9 ± 0.1
	CON-EEp/F	4	98.8 ± 18.0	56.5 ± 11.4	43.3 ± 7.5	57.0 ± 2.8	43.0 ± 2.7	0.8 ± 0.1
	P-value		NS	NS	NS	NS	NS	NS

TRT, treatment.
