# Supplementary material for: Oviduct and endometrial epithelium improve in vitro produced bovine embryo developmental kinetics
Source: Reproduction. 2024 Apr 17;167(5):e240008. doi: 10.1530/REP-24-0008 (PMC11056959; doi:10.1530/REP-24-0008)
Supplement: Supplementary Table 6. Data related to expanded blastocyst (ExB) trophectoderm (TE) and inner cell mass (ICM) cell number and percentage as well as the total cell number and the ICM:TE ratio for statistical analysis (SA) 1, 2 and 3 of the study. [file supplementary_table_6.pdf]

Supplementary Table 6. Data related to expanded blastocyst (ExB) trophectoderm (TE) and inner cell mass (ICM) cell number and percentage as well as the total cell number and the ICM:TE ratio for statistical analysis (SA) 1, 2 and 3 of the study.

SA	TRT	ExB (n)	Total Cell Number	TE Cell Number	ICM Cell Number	TE Cell %	ICM Cell %	ICM:TE Ratio
SA1	CON-CON	4	144.6 ± 19.0	90.3 ± 13.0	54.1 ± 8.6	62.0 ± 3.5	38.0 ± 3.5	0.6 ± 0.1
	OEp+	23	149.0 ± 11.6	92.5 ± 6.8	57.0 ± 5.8	61.6 ± 1.6	38.4 ± 1.6	0.6 ± 0.1
	CON+	15	128.6 ± 12.5	79.0 ± 7.7	49.9 ± 6.1	61.8 ± 1.9	38.2 ± 1.9	0.6 ± 0.0
	P-value	NA	NS	NS	NS	NS	NS	NS
SA2	CON-CON	4	143.9 ± 20.2	90.3 ± 13.7	53.8 ± 9.0	62.0 ± 3.5	40.0 ± 3.5	0.6 ± 0.1
	+EEp	19	128.9 ± 14.3	79.2 ± 8.7	50.7 ± 6.6	60.9 ± 1.8	39.1 ± 1.8	0.6 ± 0.1
	+EEp/F	19	149.2 ± 13.7	92.8 ± 8.3	56.6 ± 6.3	62.3 ± 1.7	37.7 ± 1.7	0.6 ± 0.1
	P-value	NA	NS	NS	NS	NS	NS	NS
SA3	CON-CON	4	144.2 ± 19.8	90.4 ± 13.6	54.0 ± 8.7	62.0 ± 3.4	38.0 ± 3.4	0.6 ± 0.1
	OEp-EEp	9	144.1 ± 16.1	87.7 ± 10.4	57.7 ± 7.2	59.2 ± 2.3	40.8 ± 2.3	0.7 ± 0.1
	OEp-EEp/F	14	147.5 ± 14.4	93.3 ± 8.9	54.9 ± 6.5	63.1 ± 1.9	36.9 ± 1.9	0.6 ± 0.1
	CON-EEp	10	114.1 ± 15.8	71.8 ± 10.1	43.5 ± 7.1	62.6 ± 2.2	37.4 ± 2.2	0.6 ± 0.1
	CON-EEp/F	5	152.6 ± 18.3	91.2 ± 12.5	60.5 ± 8.1	60.4 ± 3.1	39.6 ± 3.1	0.7 ± 0.1
	P-value		NS	NS	NS	NS	NS	NS

TRT, treatment.
